# Macrophage migration inhibitory factor promotes resistance to MEK blockade in KRAS mutant colorectal cancer cells

**DOI:** 10.1002/1878-0261.12345

**Published:** 2018-07-11

**Authors:** Seul‐Ki Cheon, Hwang‐Phill Kim, Ye‐Lim Park, Jee‐Eun Jang, Yoojoo Lim, Sang‐Hyun Song, Sae‐Won Han, Tae‐You Kim

**Affiliations:** ^1^ Department of Molecular Medicine & Biopharmaceutical Sciences Graduate School of Convergence Science and Technology Seoul National University Korea; ^2^ Cancer Research Institute Seoul National University Korea; ^3^ Department of Internal Medicine Seoul National University Hospital Korea

**Keywords:** colorectal cancer, feedback mechanism, KRAS, MIF, refametinib

## Abstract

Although MEK blockade has been highlighted as a promising antitumor drug, it has poor clinical efficacy in KRAS mutant colorectal cancer (CRC). Several feedback systems have been described in which inhibition of one intracellular pathway leads to activation of a parallel signaling pathway, thereby decreasing the effectiveness of single‐MEK targeted therapies. Here, we investigated a bypass mechanism of resistance to MEK inhibition in KRAS CRC. We found that KRAS mutant CRC cells with refametinib, MEK inhibitor, induced MIF secretion and resulted in activation of STAT3 and MAPK. MIF knockdown by siRNA restored sensitivity to refametinib in KRAS mutant cells. In addition, combination with refametinib and 4‐IPP, a MIF inhibitor, effectively reduced the activity of STAT3 and MAPK, more than single‐agent treatment. As a result, combined therapy was found to exhibit a synergistic growth inhibitory effect against refametinib‐resistant cells by inhibition of MIF activation. These results reveal that MIF‐induced STAT3 and MAPK activation evoked an intrinsic resistance to refametinib. Our results provide the basis for a rational combination strategy against KRAS mutant colorectal cancers, predicated on the understanding of cross talk between the MEK and MIF pathways.

**Abbreviations**
CRC: colorectal cancer
EGF: epidermal growth factor
ERK: extracellular signal‐regulated kinase
IL: interleukin
MEK: mitogen‐activated protein kinase
MIF: macrophage inhibitory factor
RTK: receptor tyrosine kinase
STAT3: signal transducer and activator of transcription 3

## 1 Introduction

The mitogen‐activated protein kinase (MAPK) pathway plays a role in various cellular functions including cell development, differentiation, proliferation, and angiogenesis. This pathway is induced through a ligand binding to a receptor, which activates kinases KRAS–BRAF–MEK–ERK in a continuative order. Among these kinases, KRAS is a clearly important component in the pathogenesis of cancer. Most KRAS mutations are positioned in codons 12, 13, and 61, leading to uncontrolled regulation through a consistently activated signaling cascade. Aberrant cell growth is induced through uncontrolled cell regulation that promotes tumorigenesis. In colorectal cancer, KRAS mutations have been detected in 40% of cases. For this reason, therapeutic approaches to overcome KRAS‐driven cancer have been studied for several decades (Zhang and Cheong, 2016). Despite efforts to target KRAS mutant CRC, none of them have succeeded in significantly improving antitumor effects.

mitogen‐activated protein kinase is an essential and promising drug target because it is a direct RAF downstream kinase and the only substrate of ERK1/2 (Akinleye *et al*., 2013; Shaul and Seger, 2007). The molecule possesses an allosteric pocket structure adjacent to, but separate from, the ATP‐binding site. Because the allosteric binding site combines with a MEK inhibitor, it stabilizes an inactive conformation of MEK1 and MEK2, and consequently inhibits ERK signaling. MEK inhibitors, such as refametinib, cobimetinib, and selumetinib, have been investigated in both cell lines and human xenograft models (Chang *et al*., 2010; Iverson *et al*., 2009). Among them, refametinib is the only cyclopropane‐1‐sulfonamide derivative and exhibits highly selective allosteric inhibition of MEK1/2 (Iverson *et al*., 2009). In a phase I/II study of patients with advanced solid tumors, refametinib was well tolerated with only a rash that was the most common drug‐related adverse event. Moreover, 70 patients received refametinib treatment along with sorafenib as a first‐line treatment for unresectable hepatocellular carcinoma (Lim *et al*., 2014). Among them, 65 patients were analyzed for efficacy per protocol, three had partial remission, and the median time progression was 4.1 months.

It has been shown that diverse types of tumors with BRAF and MEK mutations show sensitivity to MEK inhibitors (Arcila *et al*., 2015; Gilmartin *et al*., 2011; Hatzivassiliou *et al*., 2013; Solit *et al*., 2006). However, there are several reports that have investigated resistance mechanisms to MEK inhibitors. Some reports have shown a negative feedback loop through DUSPG expression of downstream ERK and induction of receptor tyrosine kinase (RTK) ligands such as interleukin (IL)‐6, Nogo‐66 receptor 1, and hepatocyte growth factor (Cheng *et al*., 2015; Furukawa *et al*., 2003; Hatzivassiliou *et al*., 2012; Lee *et al*., 2014; Wang *et al*., 2011). In addition, STAT3 activation or ERK rebound is related to resistance to MEK inhibitors in cancer (Corcoran *et al*., 2011; Lee *et al*., 2014; Zhao *et al*., 2015). Therefore, exploring mediators of the feedback mechanism may be promising to eradicate resistance to MEK inhibitors. In particular, it has been reported that KRAS‐mutated tumors show partial sensitivity or resistance to MEK inhibitors (Adjei *et al*., 2008; Lee *et al*., 2014; Sun *et al*., 2014). Although efforts have been made to investigate the mechanism of resistance to MEK blockade, it has not been clearly defined in KRAS‐driven CRCs.

Macrophage migration inhibitory factor (MIF) is a pleiotropic multifunctional cytokine. A number of studies suggest that MIF may be involved in processes regulating cell proliferation, tumor angiogenesis, and metastasis through activation of STAT3, ERK, and phosphatidylinositide 3‐kinase/AKT pathways (Lue *et al*., 2002, 2007; Lv *et al*., 2016; Ohta *et al*., 2012; Shimizu *et al*., 1999). Blockade of expression by knockout or stable RNA interference decreases tumor growth in mouse models of CRC, pancreatic cancer, and lung cancer (Costa‐Silva *et al*., 2015; Mawhinney *et al*., 2015; Ogawa *et al*., 2000). In particular, MIF activation confers chemotherapeutic resistance, and its inhibition through MIF inhibitor 4‐IPP reverses chemotherapy resistance in SCCVII squamous carcinoma cells (Kindt *et al*., 2013; Yu *et al*., 2006).

In this study, we investigated whether MIF induced by MEK blockade evokes the intrinsic resistance mechanism of KRAS‐driven CRC. Our results showed that refametinib increased MIF expression in KRAS mutant CRC cells. We also found that inhibition of MIF by 4‐IPP suppressed cell proliferation and induced apoptosis by activating caspase 3 and downregulating cyclin D1.

## 2 Materials and methods

### 2.1 Cell lines and reagents

Human CRC cell lines were obtained from the Korean Cell Line Bank (Seoul, Korea) or American Type Culture Collection (Manassas, VA, USA; Ku and Park, 2005). Cells were cultured at 37 °C in a humidified atmosphere with 5% CO2 and grown in RPMI‐1640 or Dulbecco's modified Eagle's medium containing 10% fetal bovine serum (Gibco, Carlsbad, CA, USA) and 50 μg·mL^−1^ gentamicin. Refametinib (Bay 86‐9766) was kindly provided by Bayer. 4‐IPP was purchased from Selleck Chemicals (Houston, TX, USA). Stock solutions were prepared in dimethyl sulfoxide (DMSO) and stored at −20 °C.

### 2.2 Growth inhibition assays

The viability of cells was assessed by MTT assays (Sigma‐Aldrich, St Louis, MO, USA). A total of 2 × 10^3^–1.2 × 10^4^ cells were seeded in 96‐well plates, incubated for 24 h, and then treated for 72 h with the indicated drugs at 37 °C. After the treatments, MTT solution was added to each well, followed by incubation for 4 h at 37 °C. The medium was removed, and then, DMSO was added, followed by thorough mixing for 10 min at room temperature. Cell viability was determined by measuring absorbance at 540 nm using a VersaMax microplate reader (Molecular Devices, Sunnyvale, CA, USA). The concentrations of drugs required to inhibit cell growth by 50% (IC_50_) were determined using GraphPad Prism (La Jolla, CA, USA). Six replicate wells were used for each analysis, and at least three independent experiments were conducted. The data from replicate wells are presented as the mean number of the remaining cells with 95% confidence intervals.

### 2.3 Protein extraction and western blotting

Antibodies against p‐STAT3 (pY705), p‐AKT (pS473), p‐ERK1/2 (Thr202/Tyr204), p‐MEK1/2 (pS221/221), p‐BRAF (pS445), AKT, ERK1/2, MEK1/2, cyclin D, cyclin E, p‐S6 (pS240,244), Bcl‐2, Bcl‐XL, Bim, and active caspase 3 were purchased from Cell Signaling Technology (Beverley, MA, USA). An anti‐p27 antibody was purchased from Santa Cruz Biotechnology (Santa Cruz, CA, USA). The anti‐MIF antibody was purchased from R&D Systems (Minneapolis, MN, USA). Subconfluent cells (70–80%) were used for protein analyses. The cells were treated under various conditions as described. Cells were lysed in RIPA buffer on ice for 15 min (50 mmol·L^−1^ Tris/HCl, pH 7.5, 1% NP‐40, 0.1% Na deoxycholate, 150 mmol·L^−1^ NaCl, 0.1 mmol·L^−1^ aprotinin, 0.1 mmol·L^−1^ leupeptin, 0.1 mmol·L^−1^ pepstatin A, 50 mmol·L^−1^ NaF, 1 mmol·L^−1^ sodium pyrophosphate, 1 mmol·L^−1^ sodium vanadate, 1 mmol·L^−1^ nitrophenolphosphate, 1 mmol·L^−1^ benzamidine, and 0.1 mmol·L^−1^ PMSF) and centrifuged at 12 000 ***g*** for 20 min. Samples containing equal amounts of total protein were resolved in SDS polyacrylamide denaturing gels, transferred to nitrocellulose membranes, and probed with antibodies. Detection was performed using an enhanced chemiluminescence system (Amersham Pharmacia Biotech, Buckinghamshire, UK).

### 2.4 Cell cycle analysis

For cell cycle analysis, cells were washed twice in phosphate‐buffered saline (PBS), fixed in 70% ethanol, and stored at −20 °C until analysis. Before the analysis, cell suspensions were rinsed with PBS, digested with RNase A (50 mg·mL^−1^) for 15 min at 37 °C, and stained with propidium iodide (50 mg·mL^−1^). The DNA content (10 000 cells/experimental group) was determined using a FACSCalibur flow cytometer (Becton Dickinson Biosciences, San Jose, CA, USA) with the ModFit LT program (Verity Software House Inc, Topsham, ME, USA) as described previously (Kim *et al*., 2009).

### 2.5 Real‐time RT‐PCR

Total RNA was extracted with TRI reagent (Molecular Research Center, Cincinnati, OH, USA) as described previously (Han *et al*., 2014). cDNA was synthesized from 1 mg total RNA with ImProm‐II™ reverse transcriptase (Promega Corporation, Madison, WI, USA) using random hexamers. RT‐PCR was performed using SYBR Green I (Molecular Probe, Eugene, OR, USA) and an iCycler IQ detection system (Bio‐Rad Laboratories, Hercules, CA, USA). All reactions were performed in duplicate. The primers used for RT‐PCR were as follows: MIF, forward primer 5′‐ATCGTAAACACCAACGTGCC‐3′ and reverse primer 5′‐TTGCTGTAGGAGCGGTTCTG‐3′; and 18S rRNA, forward primer 5′‐AAACGGCTACCACATCCA AG‐3′ and reverse primer 5′‐CCTCCAATGGATCCTCGTTA‐3′.

### 2.6 Enzyme‐linked immunosorbent assay (ELISA)

An ELISA for MIF was used to measure the secreted cytokine by KRAS mutant CRC cells. The cells were incubated with or without refametinib (1 μm) in serum‐free medium for 48 h. Culture supernatants were collected at the indicated times, and the amounts of secreted MIF in the supernatants were quantified using a commercially available ELISA kit (R&D Systems, Minneapolis, MN, USA).

### 2.7 Conditioned medium preparation

To prepare conditioned medium (CM), HCT116 cells were seeded in a 150‐mm culture dish. The cells were incubated in serum‐free RPMI for 48 h to produce CM. The CM was collected, centrifuged at 500 ***g*** for 5 min, filtered through a 0.2‐μm filter to remove cellular debris, and finally stored at −80 °C until use.

### 2.8 Plasmid constructs and transfection

Macrophage inhibitory factor cDNA was purchased from the Korea Human Gene Bank (Daejeon, Korea). The primers used for cloning were as follows: MIF, forward primer 5′‐GGCGAATTCATGCCGATGTTCATCGTAAACA‐3′ (including a 5′ EcoRI site) and reverse primer 5′‐GCCCTCGAGTTAGGCGAAGGTGGAGTTGTTC‐3′ (including a 5′ XhoI site). The amplified fragments were cloned into the pCMV‐Tag2B simple vector (Addgene, Cambridge, MA, USA). sgRNA targeting MIF were designed using the genscript online tool (http://www.genscript.com). The following sgRNA sequences were used: forward primer 5′‐CACCGGAGGAACCCGTCCGGCACGG‐3′ and reverse primer 5′‐AAACCCGTGCCGGACGGGTTCCTCC‐3′. Oligos were annealed and cloned into the lentiCRISPR2 vector (Addgene, Cambridge, MA, USA) using a standard BsmBI protocol. All resulting plasmids were verified by Sanger sequencing.

Transient transfection was conducted using Lipofectamine 2000 (Invitrogen, Carlsbad, CA, USA), according to the protocol suggested by the manufacturer. The LentiCRISPR2 MIF knockout construct was transfected into the HCT116 cell line using Lipofectamine 2000 to generate stable cell lines through selection with puromycin.

### 2.9 Small interfering RNA knockdown

Small interfering RNA (siRNA) against MIF was purchased from Mbiotech (Seoul, Korea). Cells were transfected with siRNA (50 nmol·L^−1^) twice every 2 days using G‐Fectin (Genolution, Seoul, Korea) in accordance with the manufacturer's instructions. Cell lysates were harvested after 48 h of drug treatment.

### 2.10 Colony formation assay

For each cell line, 500 cells were seeded in 6‐well plates in duplicate. The medium was changed every 2 days. For treatment with MIF and refametinib, MIF (100 ng·mL^−1^) and refametinib (1 μm) were added to the medium at each medium change. Cells were grown for 11 days at 37 °C with 5% CO2. The cells were washed with ice‐cold PBS and stained with 0.5% crystal violet in 25% methanol.

### 2.11 Calculation of the combination index

The combination index (CI), which was used for data analysis of two drug combinations, was calculated according to the Chou–Talalay method (Chou and Talalay, 1984). CI < 1, CI = 1, and CI > 1 indicate synergism, an additive effect, and antagonism, respectively. The efficacy of a combination of refametinib with 4‐IPP was determined. The additive, synergistic, or antagonistic effects of the combination of refametinib with 4‐IPP were calculated for each administration regimen using Calcusyn software (Biosoft, Cambridge, UK).

### 2.12 Annexin V‐binding assay for apoptosis

Cells were collected after 48 h of drug treatment. Apoptosis rate was assessed using the annexin V‐binding assay according to the protocol of the manufacturer (BD Biosciences, CA, USA). Cells were stained with propidium iodide and annexin V for 15 min at room temperature in the dark and then analyzed by flow cytometry.

### 2.13 Plasma samples

Blood was collected from patients with colorectal cancer at Seoul National University Hospital. From patients who agreed to voluntarily donate their blood for research purposes, 4–6 mL whole blood was collected into EDTA tubes during phlebotomy. Plasma was separated by centrifugation with Ficoll solution at 840 ***g*** for 15 min and transferred into microcentrifuge tubes. Then, the plasma was centrifuged at 16 000 ***g*** for 10 min to remove cell debris. The supernatant was stored at −80 °C until use.

### 2.14 TCGA data analysis

Gene expression measurements were obtained by downloading the ‘Colorectal Adenocarcinoma (TCGA, provisional)’ dataset using cBioPortal (http://www.cbioportal.org/, version 1.8.1) from The Cancer Genome Atlas (https://cancergenome.nih.gov/). The dataset contained microarray measurements for CRC patients. Gene expression levels in colorectal adenocarcinoma were represented as *z*‐scores.

### 2.15 Statistical analysis

All experiments were conducted in duplicate or triplicate, with at least two biological replicates. All data are expressed as the mean ± standard deviation. Statistical significance was calculated using Prism 7.01 software (GraphPad, San Diego, CA, USA). Comparisons between groups were analyzed by the Mann–Whitney t‐test or unpaired t‐test. The Kolmogorov–Smirnov test was used for TCGA analysis. A value of *P *<* *0.05 was considered to be significant.

## 3 Results

### 3.1 Refametinib disrupts the MAPK pathway and induces apoptosis and G1 arrest

**Figure 1:**
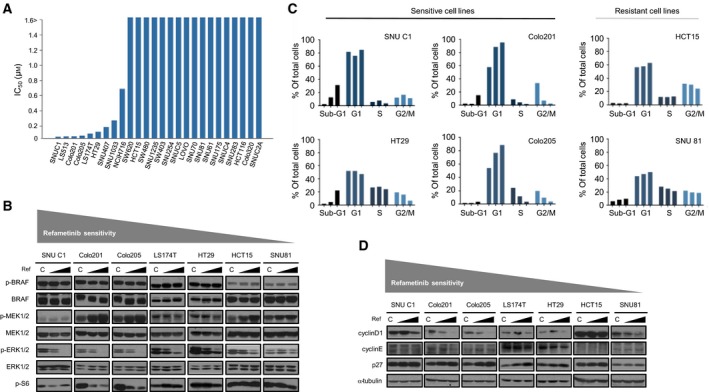
Sensitivity to refametinib varies in 26 CRC cell lines. (A) Cell proliferation assays of a panel of 26 CRC cell lines. Cells were exposed to increasing concentrations of refametinib for 72 h and evaluated for proliferation by MTT assays as described in the Materials and methods section. (B) Western blot analyses of downstream effector proteins of the MAPK signaling pathway. The panel of CRC cell lines was treated with or without refametinib (1 μm) for 72 h. α‐Tubulin served as a loading control. (C) Cell cycle distribution analysis. At 72 h after treatment with various doses of refametinib (0, 0.1, and 1 μm), analysis of the cell cycle distribution was conducted by propidium iodide staining. (D) Western blot analyses of cell cycle effector proteins in CRC cell lines after treatment with refametinib (0, 0.1, and 1 μm) for 72 h. α‐Tubulin served as a loading control.

To investigate the effects of refametinib in CRC cell lines, MTT assays were conducted to determine the difference in cellular viability of a panel of 26 CRC cell lines (Fig. 1A). Nine of 26 CRC cell lines with an IC_50_ of <1 μm were refametinib‐sensitive, whereas the other cell lines were resistant to this compound with an IC_50_ of >1 μm. Among them, we used SNUC1, Colo201, Colo205, LS174T, and HT29 cells as sensitive cell lines and HCT15 and SNU81 as resistant cell lines to refametinib. After validation of drug sensitivity in CRC cells, we characterized genetic alterations of sensitive and resistant cell lines (Table S1). Among sensitive cell lines, Colo201, Colo205, HT29, and LS174T had a BRAF mutation that is known to improve the antitumor effect of a MEK inhibitor in melanoma with a BRAF mutation (Solit *et al*., 2006). SNUC1 cells, which had the F53L mutation in MEK1, showed the most hypersensitivity to refametinib treatment. This mutation triggered dependence on the MAPK pathway as a potential therapeutic target of the MEK inhibitor (Arcila *et al*., 2015). We also found that nine of 17 resistant cell lines had a KRAS mutation (data not shown). Further investigation into the cellular effect by western blot analysis revealed that the p‐ERK1/2 level was significantly reduced upon refametinib treatment of sensitive cell lines (Fig. 1B). However, there were no remarkable changes in resistant cell lines. Levels of p‐BRAF were not changed in both types of cell lines. MEK inhibition also led to an increase in p‐MEK1/2, which correlates with the known characteristic of refametinib (Hatzivassiliou *et al*., 2013). Next, to investigate the mechanisms of cell death, we analyzed the cell cycle in the two types of cell lines by flow cytometry (Fig. 1C). Refametinib treatment showed dose‐dependent induction of apoptosis in SNUC1 and HT29 cells. Moreover, G1 arrest was induced with a reduction in Colo201 and Colo205 cells in S phase. We also validated these results through reduction in cyclin D1 and induction of p27, which are markers of the cell cycle, in five sensitive cell lines by western blot analysis. However, there were no significant changes in resistant cell lines (Fig. 1D). We also analyzed apoptotic molecules and found induction of active caspase 3 and Bim, and a reduction in Bcl‐2 in sensitive cell lines (Fig. S1). Taken together, we found that the MEK inhibitor improved tumor regression in the presence of BRAF and MEK1 mutations.

### 3.2 Increased MIF expression is associated with refametinib‐induced resistance in CRC cells

**Figure 2:**
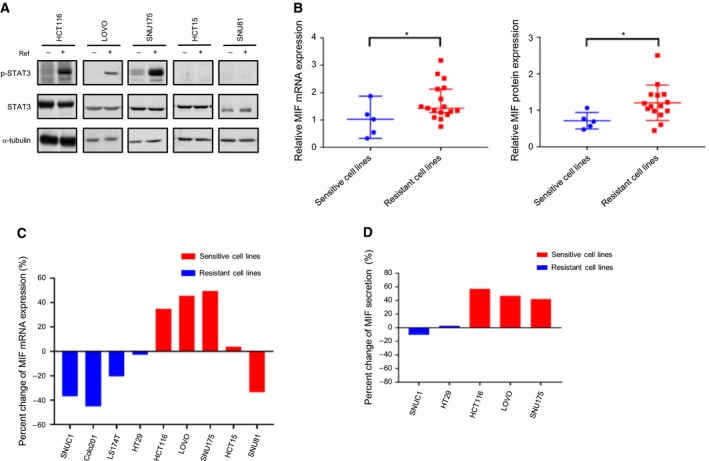
MIF expression is high in refametinib‐resistant cell lines. (A) KRAS mutant CRC cell lines were treated with or without refametinib (1 μm) for 48 h. p‐STAT3 and total STAT3 levels were determined by western blotting. α‐Tubulin served as a loading control. (B) Relative expression levels of MIF mRNA in sensitive and resistant cells, as determined by quantitative RT‐PCR, and basal expression levels of MIF protein, as determined by quantification of total MIF proteins normalized to total 18S mRNA and α‐tubulin, respectively, using ImageJ freeware (*P <* *0.05, Mann–Whitney t‐test, *P <* *0.05, unpaired t‐test). (C) The panel of human CRC cell lines was treated with refametinib (1 μm) for 48 h after 24 h of serum starvation. MIF mRNA expression was normalized to 18S mRNA, as evaluated by quantitative RT‐PCR. (D) Relative quantification of MIF secretion in conditioned medium from KRAS mutant CRC cell lines for 48 h with or without refametinib (1 μm) in serum‐free medium using ELISA.

To further characterize the effect of refametinib in resistant cell lines, we found out that STAT3 was activated in most of KRAS mutant cell lines by refametinib treatment (Fig. 2A). To investigate the effector that induced p‐STAT3, a RTK array was performed to investigate whether aberrant activation of receptors may have a role in the drug resistance (Fig. S2). Among 49 examined RTKs, we observed significant downregulation of epidermal growth factor (EGF) receptor following refametinib treatment. Because there was no remarkable activation based on the RTK array, we focused on relevant secreted factors including ILs and other cytokines that are known STAT3 activators (Fig. S3). These experiments identified induction of MIF and reduction in Serpin E1 as common effectors in HCT116 and SNU175 cells harboring KRAS mutations. We focused on MIF because it has been previously shown to activate STAT3 and regulate tumorigenesis (Lue *et al*., 2007; Ohta *et al*., 2012). First, to characterize MIF expression levels in sensitive and resistant cell lines, we detected expression of MIF mRNA and protein by qPCR and western blotting (Figs 2B and S4; Tables S2 and S3). We found that MIF mRNA and protein levels were significantly high in resistant cell lines. Next, we investigated the difference in MIF levels between diverse types of cell lines after refametinib treatment (Fig. 2C). Interestingly, mRNA expression of MIF was highly upregulated by refametinib in KRAS mutant cell lines, excluding HCT15 and SNU81, which did not show a remarkable increase despite high expression of MIF. As expected, depletion of MEK1 by siRNA and cobimetinib, a MEK inhibitor, also upregulated the expression of MIF (Figs S5 and S6). However, sensitive cell lines showed downregulation of MIF expression levels. To further confirm induction of secreted MIF at the protein level, an ELISA was performed using several different types of cell lines (Fig. 2D). The results showed approximately 50% increases in MIF secretion after refametinib treatment of resistant cell lines. However, sensitive cell lines did not show a change in MIF secretion. Taken together, these findings indicate that induction of MIF activates STAT3 following refametinib treatment in KRAS mutant cell lines.

### 3.3 MIF activates STAT3 and MAPK pathways and enhances drug resistance

**Figure 3:**
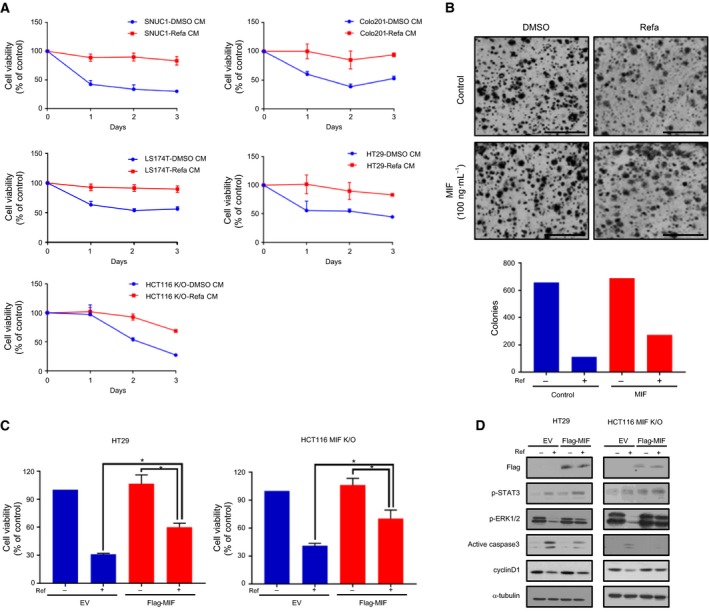
Overexpression of MIF triggers resistance to refametinib. (A) HCT116 cells were treated with or without 1 μm refametinib [DMSO‐containing conditioned medium (CM) or refametinib‐containing CM] for 72 h. CM was derived from HCT116 cells as described in the Materials and methods section. Sensitive cell lines and MIF knockout cell line (HCT116 MIF K/O) were incubated in a mixture of CM (CM to fresh media at a ratio of 1 : 4) with refametinib. Every 24 h for 3 days, cell proliferation was evaluated by MTT assays. (B) Viability of a sensitive cell line (HT29) determined by the colony formation assay. MIF (100 ng·mL^−1^) and refametinib (1 μm) were added to the medium. Bars indicate 10 mm. (C, D) Cell viability and western blot analyses of reduced apoptotic signaling pathways caused by MIF overexpression in HT29 cell line and HCT116 MIF knockout cell line (HCT116 MIF K/O). The cells were transfected for 48 h followed by 48 h of refametinib treatment (1 μm). α‐Tubulin served as a loading control (*P <* *0.05, unpaired t‐test).

To further determine whether overexpression of MIF affects drug resistance, we first investigated the possibility that secreted MIF by MEK inhibition in KRAS mutant CRCs could trigger drug resistance. Conditioned media from drug‐treated KRAS mutant CRC cells were applied to both sensitive and MIF knockout cell lines, which were treated with refametinib, and then, cell viability was determined (Figs 3A and S7). The results showed that all sensitive cell lines cultured with drug‐containing conditioned media were more resistant to refametinib than cells cultured in DMSO‐containing conditioned media. Next, recombinant MIF protein was used to evoke resistance because secreted MIF affects intrinsic drug feedback (Fig. 3B). A colony formation assay was performed by treatment with recombinant MIF to investigate the ability of single cells to grow into a colony. Incubation of HT29 cells with recombinant MIF and refametinib resulted in higher numbers of colonies than refametinib treatment alone. To validate improvement of MIF expression by the refametinib‐induced resistance mechanism, we overexpressed MIF in refametinib‐sensitive and HCT116 MIF knockout cell lines and examined whether it could induce refametinib resistance. Interestingly, overexpression of MIF increased cell viability following refametinib treatment in both cell lines (Fig. 3C). To confirm these results, we next investigated the level of protein (Fig. 3D). Western blotting indicated that the overexpressed MIF upregulated p‐STAT3 and p‐ERK1/2 and sustained p‐STAT3 and p‐ERK1/2 even after refametinib treatment. We also observed a reduction in active caspase 3 and increased cyclin D1. Taken together, MIF activation through p‐STAT3 and p‐ERK1/2 mediates resistance to the MEK inhibitor in KRAS mutant CRC cells.

### 3.4 Inhibition of MIF sensitizes KRAS mutant CRC cells to refametinib

**Figure 4:**
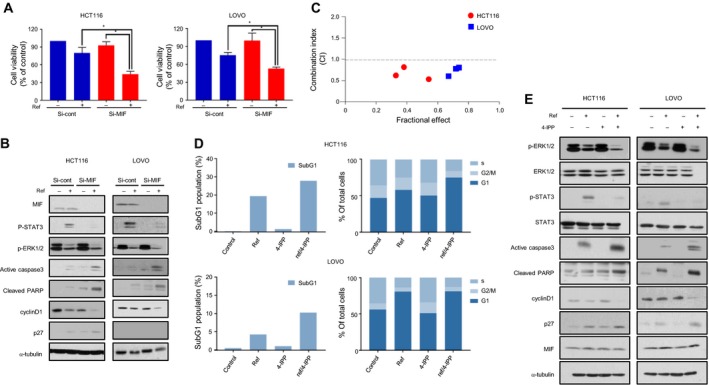
Combined treatment with MEK and MIF inhibitors is effective in KRAS mutant cell lines. (A, B) MIF siRNA were transfected into KRAS mutant cell lines for 48 h, followed by treatment with refametinib (1 μm) for 48 h and evaluation of proliferation by MTT assays and signal pathway by western blot. α‐Tubulin served as a loading control (*P <* *0.05, unpaired t‐test). (C) KRAS mutant CRC cells were incubated with refametinib and a MIF inhibitor (4‐IPP) for 48 h and then evaluated for proliferation by MTT assays at specific molar ratios of 1 : 30 and 1 : 100. CI values were calculated by the Chou–Talalay equation as described in the Materials and methods section. CI < 1 indicates synergism between the two drugs. (D, E) KRAS mutant CRC cells were incubated with refametinib (1 μm), 4‐IPP (30 μm), or their combination at the indicated concentrations for 48 h. After drug treatment, cell cycle and signal pathway were analyzed by flow cytometry and western blot. α‐Tubulin served as a loading control.

To further investigate whether MIF induces intrinsic resistance to refametinib, we examined whether inhibition by siRNA‐mediated knockdown of MIF suppressed the growth of resistant cell lines. We found that MIF knockdown following refametinib treatment resulted in a significant decrease in the viability of HCT116 and LOVO cells (Fig. 4A). To confirm these observations, we next examined protein levels of MIF, p‐STAT3, and p‐ERK1/2 (Fig. 4B). As a result, the MIF siRNA obviously decreased MIF protein expression. The results also showed reductions in p‐STAT3, p‐ERK1/2, and cyclin D1 were closely correlated with MIF suppression. On the other hand, expression of p27, active caspase 3, and cleaved PARP, was increased. Our data suggested that KRAS mutant CRC cells show induction of sensitivity to concomitant inhibition of MEK and MIF using the small molecule 4‐IPP. To test this possibility, we analyzed the CI values of two drugs and found that a drug combination exerted a synergistic effect in HCT116 and LOVO cells (Fig. 4C). This was further validated through colony formation assay (Fig. S8). Next, we analyzed the cell cycle by flow cytometry and annexin V staining to confirm this effect (Figs 4D and S9). Although refametinib and 4‐IPP alone did not affect the cell cycle, combinatorial treatment significantly increased the fraction of HCT116 cells in subG1 and G1 arrest. The LOVO cell line showed an increase in the fraction of cells in subG1, but no significant changes in G1 arrest. To investigate downstream signals of combined inhibition of MEK and MIF, we performed western blot analysis (Figs 4E and S10). HCT116 and LOVO cells exhibited increases in p‐STAT3 by refametinib treatment alone. However, combining the MIF inhibitor with refametinib abrogated the MIF‐induced p‐STAT3. Inhibition of MIF and MEK significantly reduced p‐ERK1/2 compared with single treatments. We also observed a reduction in the cyclin D1 level and induction of active caspase 3, cleaved PARP, and p27 in both cell lines. Taken together, these findings indicate that combinatorial treatment with both a MEK and MIF inhibitor effectively eliminates KRAS mutant CRC cells with refametinib‐induced resistance.

### 3.5 Comparison of MIF levels among patients harboring KRAS mutant or wild‐type tumors

**Figure 5:**
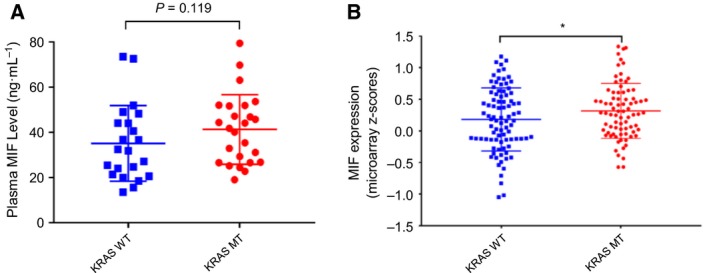
Plasma and tissue samples from CRC patients with KRAS mutation showed elevated MIF levels. (A) MIF levels in plasma of CRC patients with or without KRAS mutation as measured by an ELISA (*P =* *0.119, Mann–Whitney t‐test). (B) MIF expression *z*‐scores in KRAS wt and KRAS mutant TCGA human CRC datasets, obtained by microarray (*n* = 167) (*P <* *0.05, Kolmogorov–Smirnov test).

To expand our findings in terms of clinical significance, we measured the expression of MIF in plasma of patients with and without KRAS mutation using ELISA (Fig. 5A). The results revealed elevation of MIF in patients with a KRAS mutation (*n* = 25, median, 41.3 ng·mL^−1^; interquartile range: 19.0–79.4 ng·mL^−1^) compared with wild‐type KRAS (*n* = 22, median, 35.1 ng·mL^−1^: interquartile range: 13.5–73.5 ng·mL^−1^). This tendency was confirmed using the microarray dataset in the TCGA cohort, which includes results from a larger number of patients (Fig. 5B). We found increases in the MIF expression *z*‐scores of the KRAS‐mutated cohort (*n* = 79) compared with the wild‐type KRAS cohort (*n* = 88). Moreover, age, sex, histology, primary site, MSS/MSI status, or tumor stage did not show a significant correlation with MIF levels (data not shown). Taken together, these plasma and tissue microarray data suggest elevation of MIF expression in KRAS‐mutated patients.

## 4 Discussion

Mitogen‐activated protein kinase has been considered as a druggable target for KRAS mutant cancer. However, recent studies insist that KRAS mutant CRCs are fully resistant or partially sensitive to the antitumor effects of MEK blockade. Therefore, there is a critical need to define the resistance mechanism of MEK inhibitors for development of effective strategies to overcome diseases. The resistance mechanism can be classified into two major types: ERK‐dependent and ERK‐independent (Corcoran *et al*., 2011). RTK activation, such as overexpression of PDGFRβ and IGF1R, is included in the ERK‐independent mechanism. Mutation or overexpression of MAPK pathway components belongs to the ERK‐dependent mechanism. Here, we additionally suggest a new resistance mechanism of KRAS mutant CRCs against MEK inhibitor. In our models, STAT3 activation was observed along with MEK inhibitor treatment in KRAS mutant CRC cells. In a previous study, it was shown that MEK inhibition triggers STAT3 signaling via IL‐6 in lung cancer (Lee *et al*., 2014). However, we found that treating cells with refametinib did not increase IL‐6 (Fig. S3). In addition, DUSPG and Nogo, which are known to participate in the mechanism of resistance to MEK inhibitor, were not induced after refametinib treatment (Fig. S11). Moreover, combined MEK and STAT3 inhibition resulted in modest effects in our models (data not shown) because of activation of additional ERK‐dependent pathways. To identify activators of STAT3 and MAPK pathways upon inhibition of MEK in KRAS mutant CRC cells, we used RTK and cytokine arrays. Our data demonstrated a promising effector, MIF. Interestingly, consistent with our finding, MIF plays a role in cell regulation including survival, tumorigenesis, and activation of STAT3 and MAPK pathways. For this reason, we hypothesized that high expression and secretion levels of MIF correlate with the resistance mechanism to MEK blockade. This result was consistent with a study of the survival rate of osteosarcoma during chemotherapy (Han *et al*., 2008). Consequently, MIF secretion into the microenvironment is responsible for the autocrine effect, leading to a decrease in drug efficacy and activation of STAT3 and MAPK pathways in KRAS mutant CRCs.

Macrophage inhibitory factor is produced and secreted by MEK inhibition in KRAS mutant CRC cells, and promoting intrinsic resistance through refametinib‐conditioned medium was previously investigated to resolve resistance. Although refametinib‐conditioned medium contains various other secreted components, such as cytokines and growth factors that could impede refametinib antitumor effects, our investigation is based on a well‐defined experimental model including loss and gain of function by RNA interference, small molecule inhibitors, and overexpression such as recombinant protein treatment and MIF transfection. As a result of these experiments, we observed that MIF induced resistance to the MEK inhibitor. We also verified a similar pattern in data from public datasets on the CCLE gene expression and the GDSC drug sensitivity (Fig. S12). The refametinib/4‐IPP combination significantly induced regression of tumor growth of KRAS mutant CRCs, while the MEK inhibitor or MIF alone with small molecules did not influence tumor growth. These results were shown as reduced activity of STAT3 and ERK correlatively with MIF and MEK inhibitions. To evaluate antitumor effects by pharmacological suppression of MIF, we used 4‐IPP, a MIF inhibitor, because it is known to effectively reduce MIF activation. There are three possible mechanisms of cell activation through MIF: CD74, CXCR4, and receptor‐independent pathways. To successfully inhibit these pathways simultaneously, 4‐IPP has been used (Winner *et al*., 2008).

Macrophage inhibitory factor levels in both plasma and tissue samples were higher in CRC patients with a KRAS mutation than in those with wild‐type KRAS. Therefore, there could be a possible correlation between KRAS mutation and increased MIF levels.

Moreover, MIF is an immunomodulatory protein that attenuates immune activation and participates in the immune escape of diverse types of malignant tumors (Bach *et al*., 2008; Mittelbronn *et al*., 2011). For this reason, our findings could be consistent with several studies that combined targeted therapy with immunotherapy (Hu‐Lieskovan *et al*., 2015). This finding could provide a rationale for clinical testing of MEK and MIF blockade.

Although we investigated the relationship between MEK and MIF by various types of MEK inhibitors and silencing of MEK, we could not define the mechanism of MIF expression and secretion through MEK. Therefore, regulatory transcription factors should be further investigated for a better understanding of the relationship between MEK and MIF.

## 5 Conclusion

In conclusion, we report a new mechanism of refametinib resistance caused by MIF secretion of KRAS‐mutated CRC cells, leading to activation of bypass pathways such as STAT3 and MAPK. This study suggests a rationale to investigate the related mechanism between MIF and MEK in KRAS mutant CRCs.

## Author contributions

T‐YK, S‐KC, and H‐PK conceived the project, and T‐YK supervised the project. S‐KC and H‐PK designed and performed *in vitro* experiments. S‐KC and H‐PK analyzed the data and wrote the manuscript. Y‐LP, J‐EJ, YL, S‐HS, and S‐WH performed the validation. All authors reviewed the manuscript.

## Supporting information


**Fig. S1.** Refametinib treatment induces apoptosis of CRC cells.
**Fig. S2.** 49‐RTK array of refametinib‐treated HCT116 cells.
**Fig. S3.** Cytokines secreted from HCT116 and SNU175 cells harboring KRAS mutations after refametinib treatment.
**Fig. S4.** Analysis of basal MIF protein expression in CRC cells.
**Fig. S5.** Ch MIF in KRAS mutant CRC cells.
**Fig. S6.** MEK knockdown by siRNA induces MIF expression in KRAS mutant CRC cells.
**Fig. S7.** Validation of MIF knockout protein expression.
**Fig. S8.** Colony formation assay shows the effects of combination treatment of CRC cells with refametinib (1 μm) and 4‐IPP (30 μm).
**Fig. S9.** Results of annexin V–PI assay for HCT116 and LOVO cells treated with refametinib (1 μm) and 4‐IPP (30 μm) for 48 h.
**Fig. S10.** Western blot analysis of total MEK1/2, phosphorylated MEK1/2, and α‐tubulin after drug combination treatment for 48 h.
**Fig. S11.** Quantitative real‐time PCR data for DUSPG and Nogo‐66 receptor 1 expression in CRC cells treated with refametinib (1 μm) for 48 h.
**Fig. S12.** Correlation between the MIF mRNA expression levels and IC50 values of MEK inhibitors in CRC cells. The IC50 values for refametinib were obtained from Genomics of Drug Sensitivity in Cancer (GDSC). The MIF mRNA expression data of the cells were obtained from CCLE.
**Table S1.** Genetic alterations of CRC cells.
**Table S2.** Quantitative real‐time PCR data for MIF expression in CRC cells.
**Table S3.** Quantitative protein analysis for MIF expression in CRC cells.
