# Supplementary material for: Macrophage migration inhibitory factor promotes resistance to MEK blockade in KRAS mutant colorectal cancer cells
Source: Mol Oncol. 2018 Jul 11;12(8):1398–409. doi: 10.1002/1878-0261.12345 (PMC6068346; doi:10.1002/1878-0261.12345)
Supplement: Supplementary file 1 — Fig. S1. Refametinib treatment induces apoptosis of CRC cells. Fig. S2. 49‐RTK array of refametinib‐treated HCT116 cells. Fig. S3. Cytokines secreted from HCT116 and SNU175 cells harboring KRAS mutations after refametinib treatment. Fig. S4. Analysis of basal MIF protein expression in CRC cells. Fig. S5. Ch MIF in KRAS mutant CRC cells. Fig. S6. MEK knockdown by siRNA induces MIF expression in KRAS mutant CRC cells. Fig. S7. Validation of MIF knockout protein expression. Fig. S8. Colony formation assay shows the effects of combination treatment of CRC cells with refametinib (1 μm) and 4‐IPP (30 μm). Fig. S9. Results of annexin V–PI assay for HCT116 and LOVO cells treated with refametinib (1 μm) and 4‐IPP (30 μm) for 48 h. Fig. S10. Western blot analysis of total MEK1/2, phosphorylated MEK1/2, and α‐tubulin after drug combination treatment for 48 h. Fig. S11. Quantitative real‐time PCR data for DUSPG and Nogo‐66 receptor 1 expression in CRC cells treated with refametinib (1 μm) for 48 h. Fig. S12. Correlation between the MIF mRNA expression levels and IC50 values of MEK inhibitors in CRC cells. The IC50 values for refametinib were obtained from Genomics of Drug Sensitivity in Cancer (GDSC). The MIF mRNA expression data of the cells were obtained from CCLE. Table S1. Genetic alterations of CRC cells. Table S2. Quantitative real‐time PCR data for MIF expression in CRC cells. Table S3. Quantitative protein analysis for MIF expression in CRC cells. [file MOL2-12-1398-s001.pdf]

Supplmentary Figure S1.

Refametinib treatment induces apoptosis of CRC cells. The panel of CRC cell lines was treated with or without refametinib (1 μM) for 72 h.

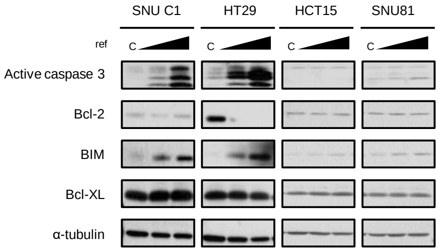

Supplementary Figure S2.

49-RTK array of refametinib-treated HCT116 cells. HCT116 cells were treated with or without refametinib (1 µM) for 48 h.

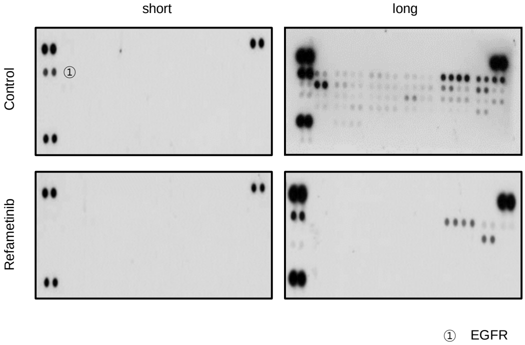

Supplementary Figure S3.

Cytokines secreted from HCT116 and SNU175 cells harboring KRAS mutations after refametinib treatment. The panel of human CRC cell lines were treated with refametinb (1 μM) for 48 h after 24 h of serum starvation.

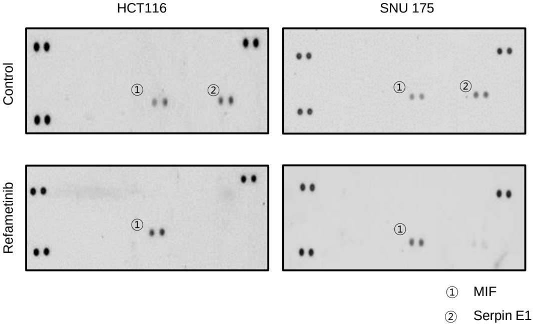

Supplementary Figure S4.

Analysis of basal MIF protein expression in CRC cells.

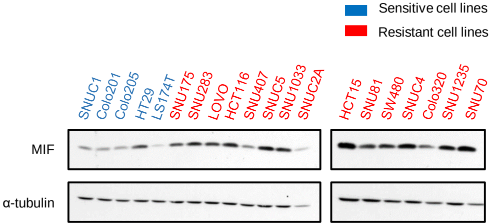

Supplementary Figure S5.

Cobimetinib activates STAT3 signaling through MIF in KRAS mutant CRC cells. Cobimetinib was treated into KRAS mutant cell lines for 48 h

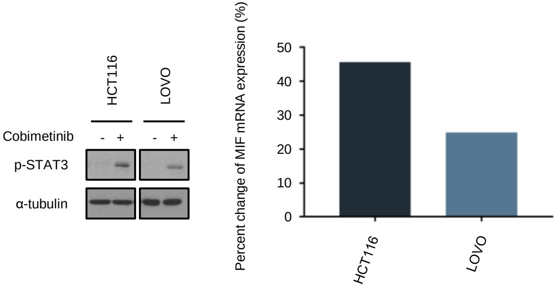

Supplementary Figure S6.

MEK knockdown by siRNAs induces MIF expression in KRAS mutant CRC cells. MEK siRNAs were transfected into KRAS mutant cell lines for 48 h.

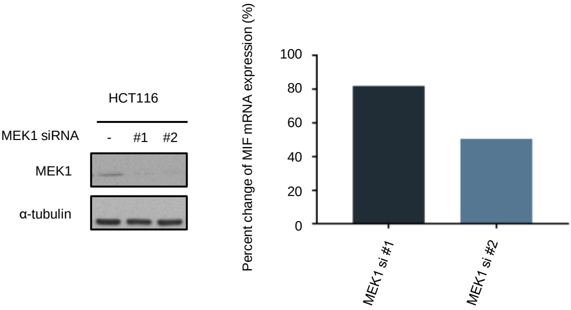

Supplementary Figure S7.

Validation of MIF knock-out protein expression.

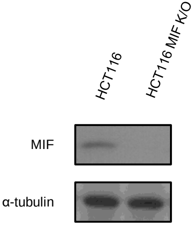

Supplementary Figure S8

Colony forming assay shows the effects of combination treatment of CRC cells with refametinib (1μM) and 4IPP (30μM).

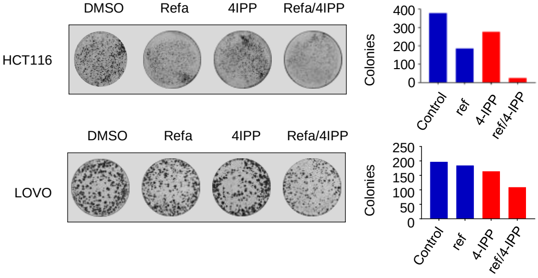

Supplementary Figure S9.

Results of annexin V-PI assay for HCT116 and LOVO cells treated with refametinib (1μM) and 4IPP (30μM) for 48 h.

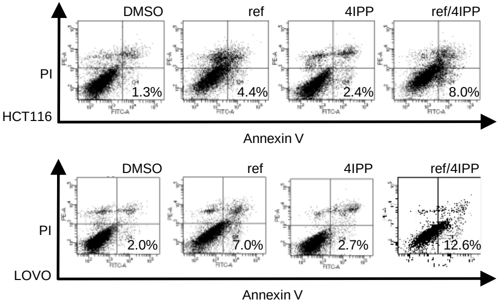

Supplementary Figure S10.

Western blot analysis of total MEK1/2, phosphorylated MEK1/2, and α-tubulin after drug combination treatment for 48 h.

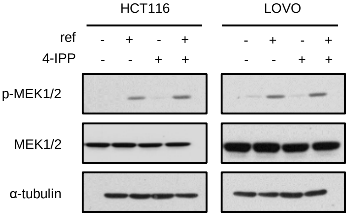

Supplementary Figure S11.

PCR data for DUSPG and Nogo-66 receptor 1 expression in CRC cells treated with refametinib (1μM) for 48 h.

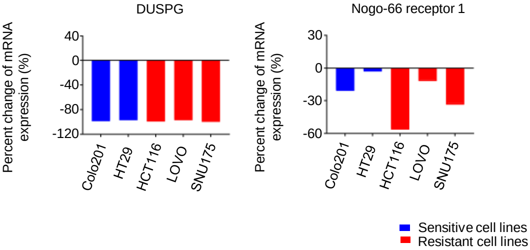

Supplementary Figure S12.

Correlation between the MIF mRNA expression levels and IC50 values of MEK inhibitors in CRC cells. The IC50 values for refametinib were obtained from Genomics of Drug Sensitivity in Cancer (GDSC). The MIF mRNA expression data of the cells were obtained from CCLE.

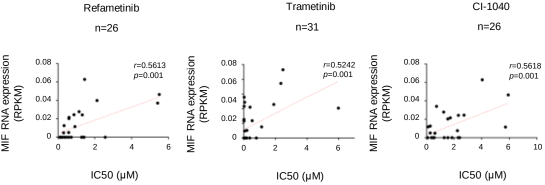

Supplementary table 1.

Genetic alterations of CRC cells.

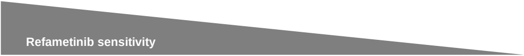

	SNUC1	COLO201	COLO205	LS174T	HT29	HCT116	LOVO	SNU175	HCT15	SNU81
KRAS	WT	WT	WT	G12D	WT	G13D	G13D	G13D	G13D	A146T
NRAS	WT	WT	WT	WT	WT	WT	WT	WT	WT	E132K
BRAF	WT	V600E	V600E	D211G	V600E , T119S	WT	WT	WT	WT	WT
RAF1	WT	WT	WT	WT	WT	WT	WT	WT	WT	WT
MAP2K1	F53L	WT	WT	WT	WT	WT	WT	WT	WT	WT
PIK3CA	WT	WT	WT	H1047R	P449T	H1047R	WT	WT	E545K	R770Q

Supplementary Table 2.

Quantitative real-time PCR data for MIF expression in CRC cells.

	cell line	ΔCt mean Value (MIF)	ΔCt Value (18s)	ΔΔCt	2^-ΔΔCt	Mean	(2^-ΔΔCt)/mean
Sensitive cell lines	SNUC1	28.05	12.47	15.58	2.04E-05	6.16279E-05	0.331278731
	colo201	21.12	8.04	13.08	0.000116		1.878672268
	colo205	23.06	8.22	14.85	3.4E-05		0.551062972
	HT29	22.50	8.79	13.72	7.43E-05		1.205917223
	LS174T	22.87	8.93	13.94	6.37E-05		1.033068807
Resistant cell lines	SNU175	21.58	8.12	13.46	8.85E-05	0.000102386	1.435527634
	SNU407	21.47	7.75	13.73	7.38E-05		1.197830695
	Lovo	21.28	7.65	13.63	7.89E-05		1.279485674
	HCT116	21.39	7.98	13.41	9.16E-05		1.486135455
	SNU283	21.49	7.93	13.57	8.24E-05		1.337025498
	SNU61	24.48	10.11	14.38	4.7E-05		0.763123477
	SNU1033	21.23	7.67	13.56	8.29E-05		1.345138012
	SNUC2A	21.68	9.03	12.65	0.000156		2.524418285
	HCT15	27.60	14.73	12.87	0.000134		2.174248008
	SNU81	22.98	9.12	13.85	6.75E-05		1.096054529
	SW480	20.68	8.12	12.56	0.000165		2.684092295
	SNU254	23.11	9.71	13.41	9.19E-05		1.491849434
	SNUC4	20.72	8.41	12.32	0.000196		3.179007704
	colo320	20.74	7.85	12.90	0.000131		2.130190635
	SNU1235	22.47	8.88	13.59	8.09E-05		1.313015057
	SNU70	23.06	9.90	13.16	0.000109		1.77043372
	SNUC5	28.90	14.96	13.94	6.38E-05		1.035412117

Supplementary Table 3.

Quantitative protein analysis for MIF expression in CRC cells.

	Area (as given by ImageJ)						
	Lane No.		Area1 (MIF)	Area2 (β-tubulin)	Area3 (MIF/β-tubulin)	Mean	Area3/Mean(Sensitive cell lines)
Sensitive cell lines	1	SNUC1	5769.92	9489.456	0.608034855	0.70816	0.858612255
	2	colo201	5835.506	8036.335	0.726140212		1.025390042
	3	colo205	4847.506	7177.213	0.675402277		0.953742483
	4	HT29	8221.749	7393.506	1.112023038		1.570299138
	5	LS174T	2723.092	6495.92	0.41920036		0.591957128
Resistant cell lines	6	SNU175	6637.92	6219.506	1.067274475	1.253439	1.507109234
	7	SNU283	8240.92	6932.213	1.188786323		1.67869736
	8	Lovo	6745.385	6837.213	0.986569381		1.393144743
	9	HCT116	8472.627	6339.506	1.336480634		1.887258013
	10	SNU407	3280.678	5461.799	0.600658867		0.848196548
	11	SNUC5	8999.627	6046.213	1.488473363		2.101888505
	12	SNU1033	7137.799	6378.213	1.119090723		1.58027949
	13	SNUC2A	1585.213	2934.092	0.540273788		0.762926158
	14	HCT15	16010.749	10951.163	1.462013578		2.06452437
	15	SNU81	9831.042	9796.042	1.003572872		1.417155546
	16	SW480	9428.163	7615.627	1.238002203		1.748195609
	17	SNUC4	12044.335	7595.627	1.585693321		2.239173803
	18	colo320	5674.799	5772.92	0.983003229		1.388108943
	19	SNU1235	9842.213	7174.042	1.371920181		1.93730256
	20	SNU70	11677.042	4126.506	2.829764939		3.995940097
